# Trends in cardiovascular mortality related to rheumatoid arthritis among U.S. adults, 1999–2023

**DOI:** 10.3389/fcvm.2026.1773739

**Published:** 2026-03-23

**Authors:** Ye Jiang, Zhichao Meng, Huiqi Yuan, Guanghua Liang, Yongping Cao

**Affiliations:** 1 Department of Orthopedics, Peking University First Hospital, Beijing, China; 2 School of Public Health, Capital Medical University, Beijing, China

**Keywords:** cardiovascular disease, CDC WONDER, disease burden, epidemiological trends, rheumatoid arthritis

## Abstract

**Introduction:**

Rheumatoid arthritis (RA) is a systemic autoimmune disease frequently accompanied by extra-articular manifestations, particularly cardiovascular disease (CVD), which contributes substantially to morbidity and mortality. Although RA is a well-recognized risk factor for CVD, population-based studies describing long-term trends in RA-related CVD mortality in the United States remain limited.

**Methods:**

Mortality data on RA-related CVD among U.S. adults aged ≥25 years from 1999 to 2023 were obtained from the Centers for Disease Control and Prevention Wide-ranging Online Data for Epidemiologic Research (CDC WONDER) database. RA and CVD were identified using International Classification of Diseases, 10th Revision (ICD-10) codes. Age-adjusted mortality rates (AAMRs, per 100,000 population) were calculated for demographic subgroups. Joinpoint regression was applied to estimate annual percent change (APC) and average annual percent change (AAPC).

**Results:**

From 1999 to 2023, a total of 63,406 deaths from RA-related CVD occurred among U.S. adults. Stratified analyses showed that female deaths were twice as common as male deaths, with an AAMR of 1.14 per 100,000 population (95% CI:1.09–1.19) compared to 0.57 per 100,000 (95% CI: 0.52–0.61) in males. Among the four U.S. Census regions, the Northeast had the lowest AAMR at 0.71 per 100,000 (95% CI: 0.61–0.82). Non-Hispanic (NH) Whites had the highest AAMR (0.97), whereas other NH groups had the lowest (0.86). Rural areas had a higher AAMR (1.11) compared to urban areas (0.85). Ischemic heart disease accounted for the largest proportion of CVD-related deaths among individuals with RA (AAMR: 0.37 per 100,000).

**Conclusion:**

This study describes long-term temporal trends and demographic disparities in RA-related CVD mortality in the United States. The recent increase in mortality warrants further investigation and may reflect multiple contributing factors, including population changes, healthcare access, and broader public health influences such as the COVID-19 pandemic. These findings identify population subgroups with disproportionately high mortality and highlight the need for further research to better understand underlying mechanisms and potential preventive strategies.

## 1 Introduction

Rheumatoid arthritis (RA) is a chronic, systemic autoimmune disorder that predominantly presents with symmetrical synovial joint inflammation and is often accompanied by extra-articular complications (1). RA affects approximately 0.46% of the global population, with prevalence differing markedly between regions and demographic groups (2, 3). In North America, the disease burden is particularly pronounced, with an estimated prevalence of 0.7% and rates as high as 2%–3% reported among some Indigenous communities (4–6). Despite advances in therapy, particularly the adoption of disease-modifying antirheumatic drugs (DMARDs), which have enhanced patient survival, RA remains associated with substantially elevated mortality relative to the general population (7, 8).

The central mechanism of RA lies in chronic systemic inflammation, which not only drives persistent joint damage and deformities but also markedly elevates the risk of comorbid chronic conditions (9, 10). Among these, CVD represents one of the most significant comorbidities (7). Epidemiological studies have shown that individuals with RA face approximately twice the risk of developing CVD compared with the general population (11). CVD encompasses a range of conditions, including heart failure, atherosclerosis, peripheral vascular disorders, cerebrovascular disease, and various structural and electrophysiological abnormalities. Inflammation plays a central role in the pathogenesis of CVD, accelerating atherosclerotic processes, promoting myocardial fibrosis, and contributing to diastolic dysfunction and heart failure with preserved ejection fraction (12).

In addition to traditional cardiovascular risk factors such as smoking, obesity, physical inactivity, hypertension, diabetes, and dyslipidemia, RA-specific factors further exacerbate the risk of CVD (13–15). These include erosive joint disease, extra-articular involvement, and persistent systemic inflammation (16). Moreover, RA therapies may exert dual effects on cardiovascular outcomes, potentially reducing inflammation-related risk while also carrying the possibility of adverse cardiovascular events. Notably, both the incidence and outcomes of RA vary substantially across sociodemographic and geographic populations. Minority groups frequently bear a disproportionate disease burden yet remain underrepresented in clinical trials and have limited access to healthcare resources (17, 18).

Given the persistent excess cardiovascular risk in RA and its uneven distribution across populations, identifying high-risk groups is crucial for targeted interventions and reducing disease burden. Accordingly, this study investigates U.S. trends in RA-related CVD mortality from 1999 to 2023, with attention to demographic patterns, regional differences, and at-risk populations.

## 2 Materials and methods

### 2.1 Data extraction

This study was a retrospective, population-based analysis using mortality data obtained from the Centers for Disease Control and Prevention (CDC) Wide-ranging Online Data for Epidemiologic Research (WONDER) platform. CDC WONDER is a publicly accessible database that provides detailed information on mortality and other public health outcomes in the United States.

The study population included decedents aged ≥25 years between 1999 and 2023 whose death certificates listed rheumatoid arthritis (RA) as either the underlying cause of death or a contributing condition. RA was identified using the International Classification of Diseases, 10th Revision (ICD-10) codes M05 (seropositive RA) and M06 (other RA), which have been widely used in prior studies to identify RA-related deaths. Cardiovascular disease (CVD) was defined using ICD-10 codes I00–I99 (19–23).

For the primary analysis, CVD was treated as the underlying cause of death (UCD), defined as the disease or injury that initiated the chain of events leading directly to death, while RA was treated as a multiple cause of death (MCD), indicating that RA was listed as a contributing condition on the death certificate. This approach was used to describe mortality patterns among individuals with RA whose deaths were attributed primarily to CVD.

Because RA may be underreported or inconsistently recorded on death certificates, some degree of misclassification is possible and should be considered when interpreting these findings. Because the data were de-identified and publicly available, institutional review board approval was not required. This study was conducted and reported in accordance with the Strengthening the Reporting of Observational Studies in Epidemiology (STROBE) guidelines (24).

After data extraction, mortality records were stratified by calendar year, sex, race/ethnicity, geographic region, and urban–rural status to evaluate temporal trends and population-level disparities. Race and ethnicity were categorized as Hispanic, non-Hispanic Black/African American, non-Hispanic White, and other non-Hispanic populations (including non-Hispanic American Indian/Alaska Native and non-Hispanic Asian or Pacific Islander). Urban–rural classification followed the National Center for Health Statistics (NCHS) scheme, which distinguishes metropolitan from non-metropolitan areas. Geographic regions were defined according to U.S. Census Bureau classifications (Northeast, Midwest, South, and West) (25, 26).

CVD subtypes were further classified as RA-related ischemic heart disease, hypertensive diseases, and cerebrovascular diseases based on ICD-10 groupings.

### 2.2 Data analysis

This study examined temporal trends and demographic as well as regional differences in RA-related CVD mortality from 1999 to 2023. Both age-adjusted mortality rates (AAMRs) per 100,000 population and crude mortality rates were estimated, with AAMRs standardized to the 2000 U.S. standard population (27). Joinpoint regression was then employed to detect significant changes in mortality trends over time and to calculate the annual percent change (APC) along with 95% confidence intervals (CIs). This log-linear regression approach allows detection of critical shifts within long-term mortality patterns. An APC significantly different from the null hypothesis was interpreted as an increasing or decreasing trend. Statistical significance was defined as a two-tailed *P* value < 0.05 (28).

## 3 Results

From 1999 to 2023, a total of 63,406 deaths from RA–related CVD were identified in the CDC WONDER database among adults aged ≥25 years. Of these, 46,663 deaths occurred in females, 20,882 in the Southern region, and 53,283 among non-Hispanic Whites. By place of residence (data available from 1999 to 2020), 43,195 deaths occurred in urban areas and 12,464 in non-urban areas. During this period, the overall average annual percent change (AAPC) in RA-related CVD mortality was −3.02 (95% CI: −3.65 to −2.39). Trend analysis revealed a pattern of sharp decline followed by a slower decrease, then a marked increase: 1999–2009 APC: −5.83 (95% CI: −6.50 to −5.16); 2009–2017 APC: −2.96 (95% CI: −4.31 to −1.59); 2017–2023 APC: 1.75 (95% CI: 0.03 to 3.49).

### 3.1 Overall and cause-specific RA-related cardiovascular mortality

**Figure 1:**
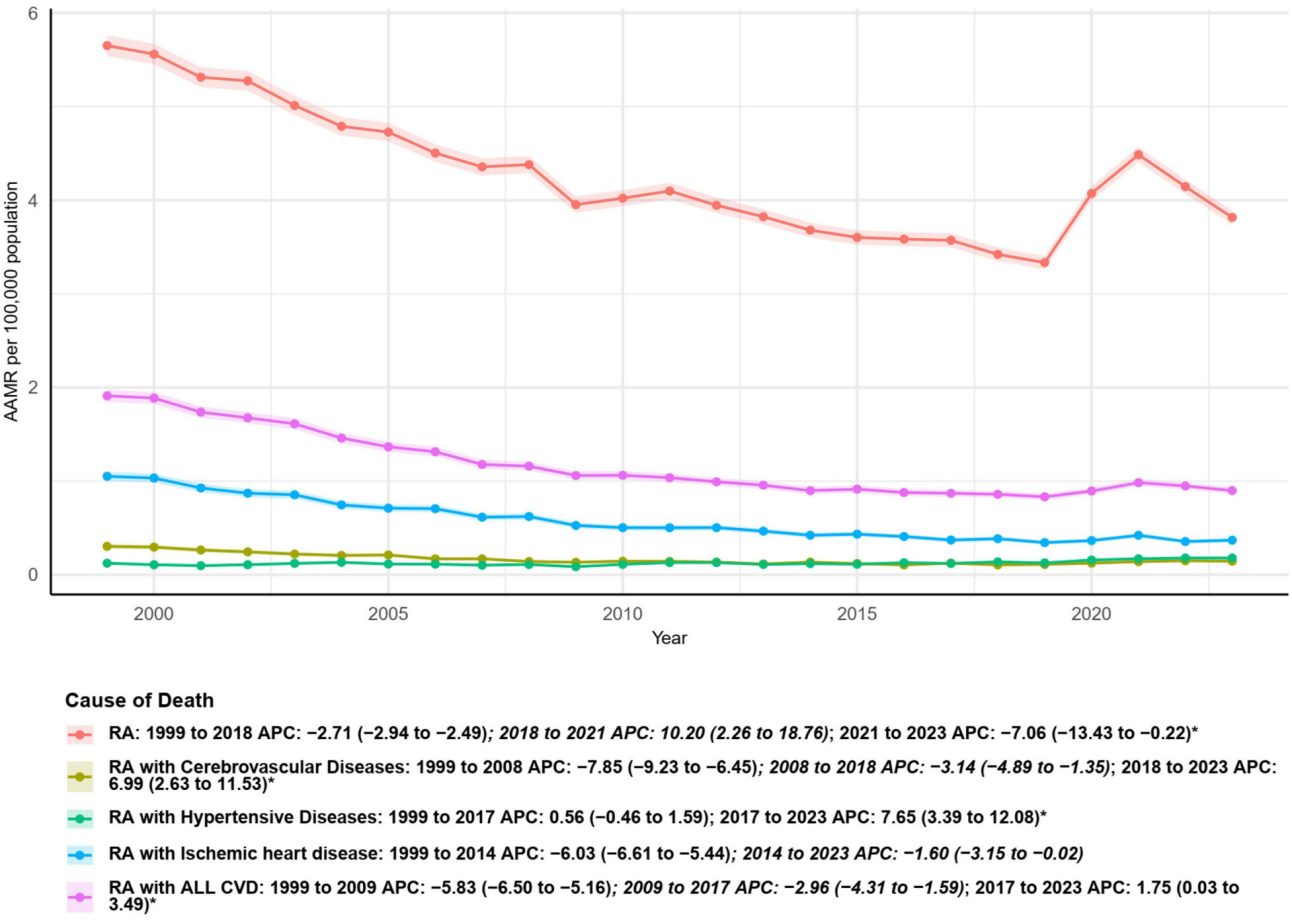
Underlying cause of death in RA patients.

Between 1999 and 2023, AAMRs (per 100,000 population) for cardiovascular mortality among decedents with RA showed an overall long-term decline, with evidence of trend reversals in recent years. For deaths where RA was the underlying cause, the mortality rate decreased significantly from 1999 to 2018 (APC −2.71, 95% confidence interval −2.94 to −2.49), then rose from 2018 to 2021 (APC 10.20, 95% confidence interval 2.26 to 18.76), and subsequently decreased again from 2021 to 2023 (APC −7.06, 95% confidence interval −13.43 to −0.22). Cause-specific analyses demonstrated heterogeneous trends. Mortality from RA with cerebrovascular diseases declined markedly from 1999 to 2008 (APC −7.85, 95% CI −9.23 to −6.45) and continued to decrease at a slower rate until 2018 (APC −3.14, 95% CI −4.89 to −1.35), before increasing significantly from 2018 to 2023 (APC 6.99, 95% CI 2.63 to 11.53). Deaths from RA with hypertensive diseases showed no significant change from 1999 to 2017 (APC 0.56, 95% CI −0.46 to 1.59), followed by a significant increase after 2017 (APC 7.65, 95% CI 3.39 to 12.08). In contrast, mortality from RA with ischemic heart disease declined continuously from 1999 to 2014 (APC −6.03, 95% CI −6.61 to −5.44) and remained on a downward trajectory thereafter, although at a slower pace (2014–2023 APC −1.60%, 95% CI −3.15 to −0.02). When considering all cardiovascular diseases combined, RA-related mortality decreased substantially from 1999 to 2009 (APC −5.83%, 95% CI −6.50 to −5.16), continued to decline until 2017, and then exhibited a modest but significant increase from 2017 to 2023 (APC 1.75%, 95% CI 0.03 to 3.49). Not only that, we analyzed RA as a potential cause of death, RA-related CV diseases, and three different types of RA-related CV diseases. Among them, the total number of deaths caused by RA as the potential cause of death was 234,407, the number of deaths related to RA-related CV diseases reached 63,406, and among them, the number of deaths related to ischemic heart disease was the highest, at 30,654 people (Figure 1; Tables 1, 2).

**Table 1:** The AAMR data for 2023 in urbanization was substituted with that of 2020, and the AAPC was calculated from 1999 to 2020.

Measure	Deaths_1999	Deaths_2023	Percent.change	AAMR_1999	AAMR_2023	AAPC (95% CI)
RA with ALL CVD	3,400	2,522	−25.82	1.91 (1.85 to 1.98)	0.90 (0.86 to 0.93)	−3.02 (−3.65 to −2.39)*
Female	2,539	1,835	−27.73	2.33 (2.24 to 2.42)	1.14 (1.09 to 1.19)	−2.83 (−3.29 to −2.36)*
Male	861	687	−20.21	1.24 (1.15 to 1.32)	0.57 (0.52 to 0.61)	−3.13 (−4.01 to −2.25)*
Northeast	586	365	−37.71	1.55 (1.43 to 1.68)	0.71 (0.63 to 0.78)	−2.93 (−3.63 to −2.22)*
Midwest	939	595	−36.63	2.21 (2.07 to 2.35)	1.01 (0.93 to 1.09)	−3.15 (−3.82 to −2.48)*
South	1,089	909	−16.53	1.75 (1.65 to 1.85)	0.87 (0.81 to 0.92)	−2.79 (−3.43 to −2.14)*
West	786	653	−16.92	2.26 (2.10 to 2.42)	1.03 (0.95 to 1.11)	−3.20 (−3.68 to −2.71)*
Hispanic	118	193	63.56	1.45 (1.18 to 1.73)	0.71 (0.61 to 0.82)	−2.51 (−3.48 to −1.52)*
NH Black	219	236	7.76	1.57 (1.36 to 1.78)	0.94 (0.82 to 1.07)	−1.81 (−2.86 to −0.76)*
NH White	3,004	1,993	−33.66	2.00 (1.93 to 2.07)	0.97 (0.92 to 1.01)	−2.88 (−3.52 to −2.24)*
NH Other	52	96	84.62	1.28 (0.95 to 1.69)	0.50 (0.40 to 0.61)	−3.03 (−3.72 to −2.33)*
Metropolitan	2,633	1,934	−26.55	1.84 (1.77 to 1.91)	0.86 (0.82 to 0.90)	−3.78 (−4.48 to −3.08)*
Nonmetropolitan	767	496	−35.33	2.26 (2.10 to 2.42)	1.11 (1.01 to 1.21)	−3.80 (−4.60 to −3.00)*
RA	10,015	10,575	5.59	5.65 (5.54 to 5.76)	3.82 (3.74 to 3.89)	−1.56 (−2.58 to −0.53)*
RA with Cerebrovascular Diseases	534	392	−26.59	0.30 (0.28 to 0.33)	0.14 (0.13 to 0.16)	−2.94 (−4.09 to −1.78)*
RA with Hypertensive Diseases	198	471	137.88	0.12 (0.11 to 0.14)	0.18 (0.16 to 0.19)	2.29 (1.08 to 3.51)*
RA with Ischemic heart disease	1,859	996	−46.42	1.05 (1.00 to 1.10)	0.37 (0.34 to 0.39)	−4.39 (−5.03 to −3.75)*

RA, All deaths in which RA was listed as a contributing factor; AAMR, age-adjusted mortality rate; CI, confidence interval; AAPC, average annual percent change; NH, non-hispanic; ischemic heart disease (ICD−10 codes I20-I25); cerebrovascular diseases (ICD-10 codes I60-I69); hypertensive diseases (ICD-10 codes I10-I15).

* AAPC values that are statistically significant.

**Table 2:** The underlying causes of death in RA patients in the United States, 1999–2023.

Year	RA		Cerebrovascular diseases		Hypertensive diseases		Ischemic heart disease		CVD	
1999	10,015	5.651 (5.541, 5.762)	534	0.303 (0.278, 0.329)	198	0.123 (0.106, 0.141)	1,859	1.051 (1.004, 1.099)	3,400	1.911 (1.847, 1.976)
2000	9,960	5.560 (5.451, 5.669)	523	0.295 (0.270, 0.321)	190	0.107 (0.091, 0.122)	1,837	1.032 (0.985, 1.080)	3,374	1.886 (1.822, 1.950)
2001	9,620	5.314 (5.208, 5.420)	477	0.264 (0.240, 0.288)	203	0.097 (0.083, 0.111)	1,663	0.926 (0.882, 0.971)	3,152	1.737 (1.676, 1.798)
2002	9,753	5.275 (5.170, 5.379)	449	0.244 (0.222, 0.267)	206	0.107 (0.092, 0.123)	1,610	0.871 (0.828, 0.914)	3,076	1.676 (1.617, 1.736)
2003	9,374	5.012 (4.910, 5.114)	431	0.221 (0.200, 0.243)	224	0.121 (0.105, 0.137)	1,606	0.854 (0.812, 0.896)	3,003	1.612 (1.555, 1.670)
2004	9,066	4.789 (4.690, 4.888)	398	0.206 (0.186, 0.227)	252	0.133 (0.116, 0.149)	1,411	0.745 (0.706, 0.784)	2,785	1.460 (1.405, 1.514)
2005	9,121	4.728 (4.630, 4.825)	405	0.211 (0.190, 0.232)	230	0.115 (0.099, 0.130)	1,365	0.711 (0.673, 0.749)	2,678	1.366 (1.314, 1.418)
2006	8,876	4.504 (4.410, 4.598)	344	0.170 (0.152, 0.188)	224	0.113 (0.098, 0.128)	1,357	0.706 (0.668, 0.743)	2,583	1.314 (1.263, 1.365)
2007	8,772	4.356 (4.265, 4.448)	347	0.170 (0.151, 0.189)	220	0.102 (0.088, 0.115)	1,219	0.615 (0.580, 0.649)	2,402	1.178 (1.130, 1.225)
2008	8,956	4.380 (4.289, 4.471)	315	0.141 (0.125, 0.157)	233	0.109 (0.095, 0.124)	1,257	0.622 (0.587, 0.656)	2,395	1.160 (1.113, 1.206)
2009	8,246	3.953 (3.867, 4.039)	301	0.133 (0.117, 0.149)	213	0.086 (0.073, 0.098)	1,116	0.526 (0.495, 0.558)	2,207	1.060 (1.015, 1.104)
2010	8,544	4.021 (3.935, 4.107)	304	0.144 (0.128, 0.161)	245	0.111 (0.097, 0.125)	1,093	0.503 (0.473, 0.533)	2,253	1.062 (1.018, 1.107)
2011	8,908	4.099 (4.013, 4.185)	304	0.143 (0.126, 0.159)	270	0.130 (0.114, 0.146)	1,083	0.502 (0.471, 0.532)	2,275	1.036 (0.993, 1.079)
2012	8,807	3.946 (3.863, 4.029)	292	0.135 (0.119, 0.150)	300	0.130 (0.115, 0.146)	1,105	0.503 (0.473, 0.533)	2,237	0.993 (0.951, 1.034)
2013	8,786	3.824 (3.743, 3.905)	277	0.115 (0.100, 0.129)	280	0.110 (0.096, 0.124)	1,065	0.465 (0.437, 0.494)	2,209	0.956 (0.916, 0.997)
2014	8,634	3.680 (3.602, 3.759)	312	0.135 (0.120, 0.150)	263	0.119 (0.104, 0.134)	1,000	0.422 (0.395, 0.449)	2,125	0.899 (0.860, 0.937)
2015	8,639	3.602 (3.525, 3.680)	304	0.119 (0.105, 0.133)	281	0.112 (0.098, 0.126)	1,027	0.433 (0.406, 0.460)	2,203	0.914 (0.875, 0.953)
2016	8,845	3.584 (3.509, 3.660)	267	0.105 (0.092, 0.118)	318	0.128 (0.114, 0.143)	1,003	0.408 (0.382, 0.434)	2,164	0.878 (0.841, 0.915)
2017	8,999	3.572 (3.497, 3.647)	329	0.124 (0.110, 0.138)	314	0.121 (0.107, 0.135)	955	0.371 (0.347, 0.395)	2,228	0.870 (0.833, 0.906)
2018	8,824	3.422 (3.350, 3.494)	294	0.105 (0.093, 0.118)	352	0.137 (0.123, 0.152)	977	0.385 (0.360, 0.410)	2,249	0.859 (0.824, 0.895)
2019	8,864	3.334 (3.264, 3.404)	311	0.110 (0.097, 0.123)	360	0.126 (0.113, 0.140)	935	0.343 (0.321, 0.366)	2,231	0.832 (0.797, 0.867)
2020	11,018	4.073 (3.996, 4.150)	340	0.124 (0.110, 0.138)	431	0.157 (0.142, 0.172)	1,022	0.365 (0.342, 0.388)	2,430	0.894 (0.858, 0.930)
2021	11,848	4.486 (4.404, 4.568)	370	0.140 (0.125, 0.154)	476	0.170 (0.154, 0.186)	1,081	0.421 (0.396, 0.447)	2,585	0.983 (0.945, 1.022)
2022	11,357	4.145 (4.068, 4.223)	404	0.149 (0.134, 0.164)	521	0.179 (0.163, 0.194)	1,012	0.355 (0.332, 0.377)	2,640	0.948 (0.912, 0.985)
2023	10,575	3.816 (3.742, 3.890)	392	0.145 (0.130, 0.159)	471	0.178 (0.162, 0.195)	996	0.369 (0.345, 0.392)	2,522	0.899 (0.863, 0.934)
Total	234,407		9,024		7,275		30,654		63,406	

### 3.2 Regional differences in RA-related cardiovascular mortality

Regional analyses revealed substantial geographic heterogeneity in AAMRs and temporal trends (Table 1). In the Northeast, mortality declined significantly from 1999 to 2015 (APC −5.25, 95% CI −5.83 to −4.67), followed by a non-significant upward trend between 2015 and 2023 (APC 1.88, 95% CI −0.09 to 3.90). The Midwest experienced sustained declines from 1999 to 2010 (APC −5.42, 95% CI −5.98 to −4.86) and from 2010 to 2016 (APC −3.26, 95% CI −5.44 to −1.03), with stabilization thereafter (2016–2023 APC 0.62, 95% CI −0.75 to 2.00). In the South, AAMRs decreased sharply from 1999 to 2014 (APC −5.49, 95% CI −6.12 to −4.84), followed by a significant increase from 2014 to 2023 (APC 1.87, 95% CI 0.35 to 3.42). The West showed the most pronounced early decline from 1999 to 2007 (APC −6.28, 95% CI −7.03 to −5.53), continued reductions until 2018, and a subsequent non-significant increase after 2018 (APC 1.05, 95% CI −0.70 to 2.83).

**Figure 2:**
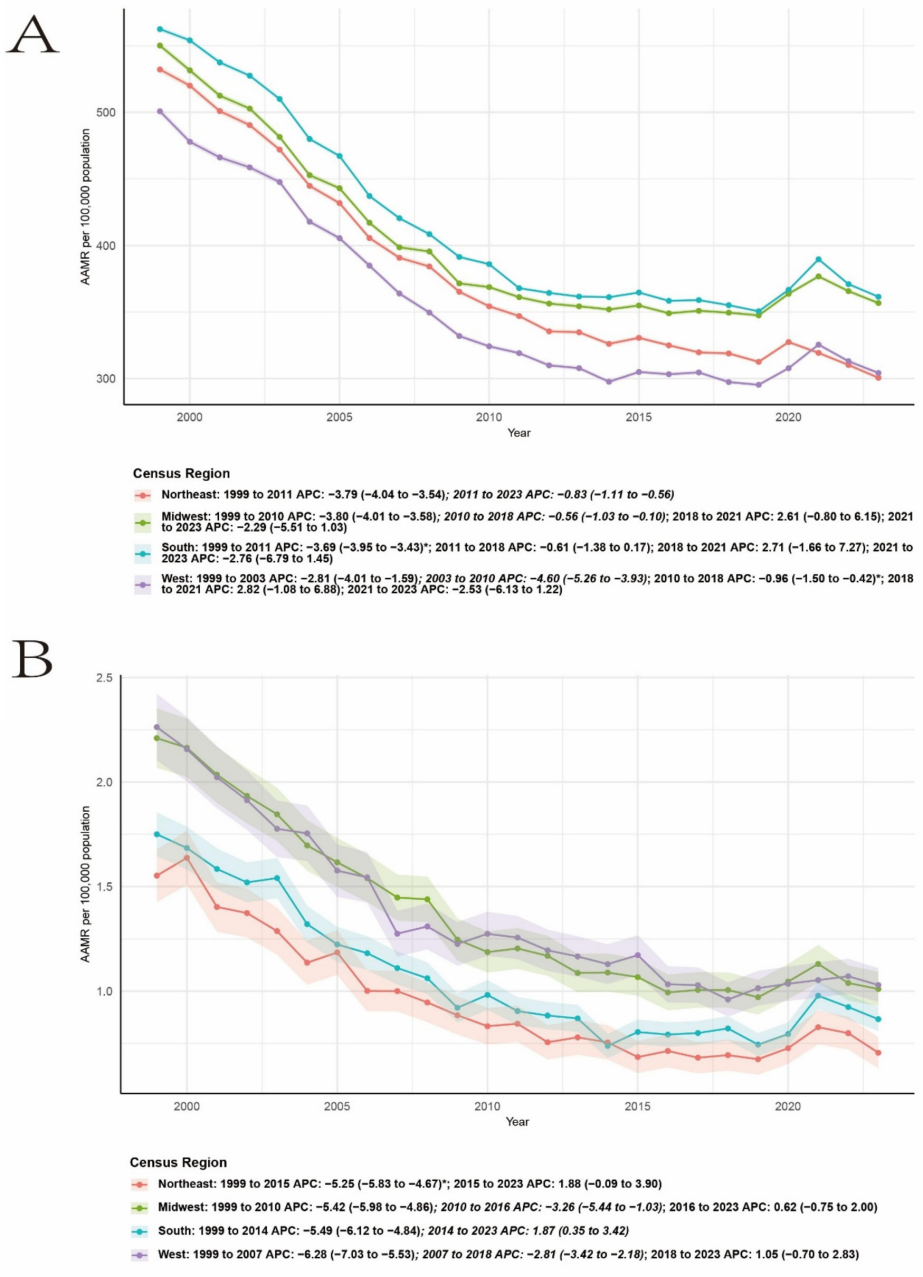
Deaths related to CVD **(A)** and RA-related CVD **(B)** among adults in the United States, stratified by region, from 1999 to 2023.

Compared with CVD related to RA, the mortality trend of CVD shows a similar pattern. Taking the northeastern region as an example, from 1999 to 2011, it showed a sharp decline trend (APC −5.49, 95% CI −6.12 to −4.84), and from 2011 to 2023, it showed a slow decline trend (Figure 2).

### 3.3 Sex-specific trends in RA-related cardiovascular mortality

**Figure 3:**
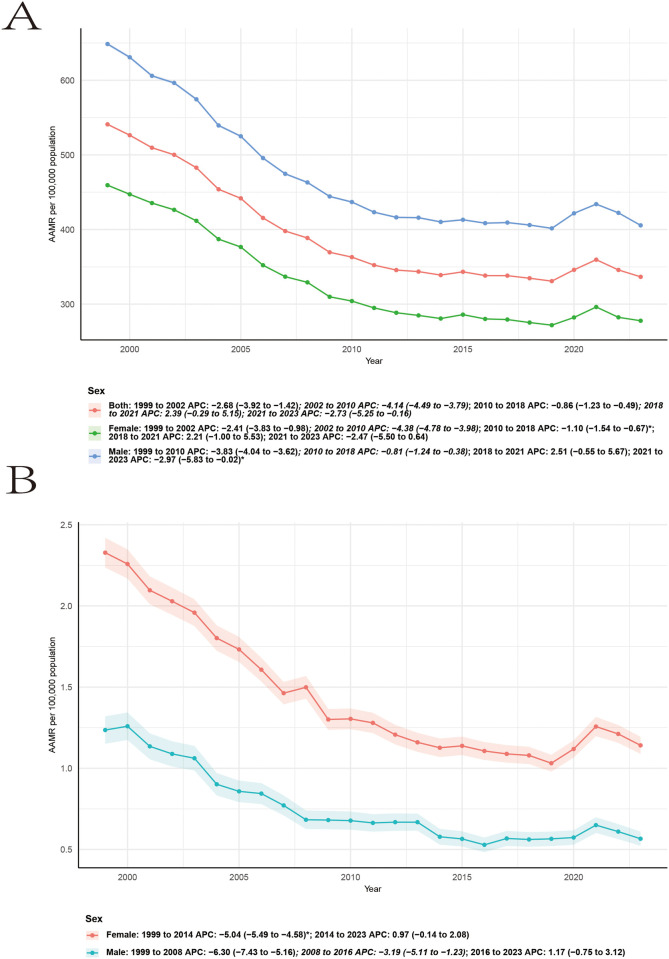
The age-adjusted mortality rate per 10,000 people for CVD **(A)** and RA-related CVD **(B)**, stratified by gender, in the United States from 1999 to 2023.

Sex-stratified analyses demonstrated parallel but quantitatively different trends in RA-related cardiovascular mortality (Figure 3B and Table 1). Among females, AAMRs declined significantly from 1999 to 2014 (APC −5.04, 95% CI −5.49 to −4.58), followed by a period of stabilization from 2014 to 2023 (APC 0.97, 95% CI −0.14 to 2.08). Among males, mortality decreased more steeply from 1999 to 2008 (APC −6.30, 95% CI −7.43 to −5.16), with continued but attenuated declines during 2008–2016 (APC −3.19, 95% CI −5.11 to −1.23). From 2016 to 2023, a non-significant increasing trend was observed (APC 1.17, 95% CI −0.75 to 3.12). Across all years, males consistently exhibited higher AAMRs than females, while both sexes showed evidence of plateauing or reversal of declining trends in the most recent period.

The mortality trend of CVD shows a pattern of decline, rise, and then decline. Taking men as an example, from 1999 to 2010, it showed a decreasing trend (APC −3.83, 95% CI −4.04 to −3.62), from 2010 to 2018, the decline trend slowed down, from 2018 to 2021, it showed an increasing trend, and from 2021 to 2023, the AAMR once again showed a decreasing trend (Figure 3).

### 3.4 Urban–rural disparities in mortality trends

**Figure 4:**
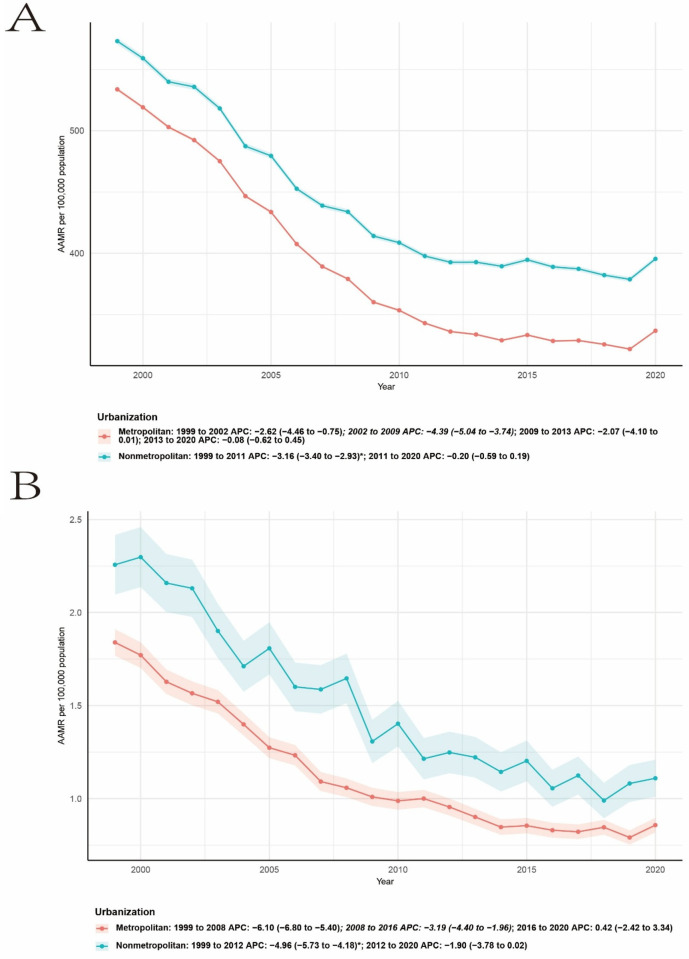
Deaths related to CVD **(A)** and RA-related CVD **(B)** among U.S. adults, stratified by urbanization, from 1999 to 2020.

Clear differences in temporal trends were observed by urbanization status (Figure 4). In metropolitan areas, RA-related cardiovascular mortality declined rapidly from 1999 to 2008 (APC −6.10, 95% CI −6.80 to −5.40), followed by a slower decline from 2008 to 2016 (APC −3.19, 95% CI −4.40 to −1.96), and subsequently stabilized from 2016 to 2020 (APC 0.42, 95% CI −2.42 to 3.34). In nonmetropolitan areas, mortality declined from 1999 to 2012 (APC −4.96, 95% CI −5.73 to −4.18), with the downward trend attenuating between 2012 and 2020 (APC −1.90, 95% CI −3.78 to 0.02). Throughout the study period, nonmetropolitan areas exhibited higher AAMRs than metropolitan areas.

The mortality trend of CVD shows a downward trend in the urban-rural regions. Taking non-metropolitan areas as an example, from 1999 to 2011 (APC −3.16, 95% CI −3.40 to −2.93), and from 2011 to 2020, the downward trend slowed down (APC −0.20, 95% CI −0.59 to −0.19) (Figure 4).

### 3.5 Racial and ethnic differences in RA-related cardiovascular mortality

**Figure 5:**
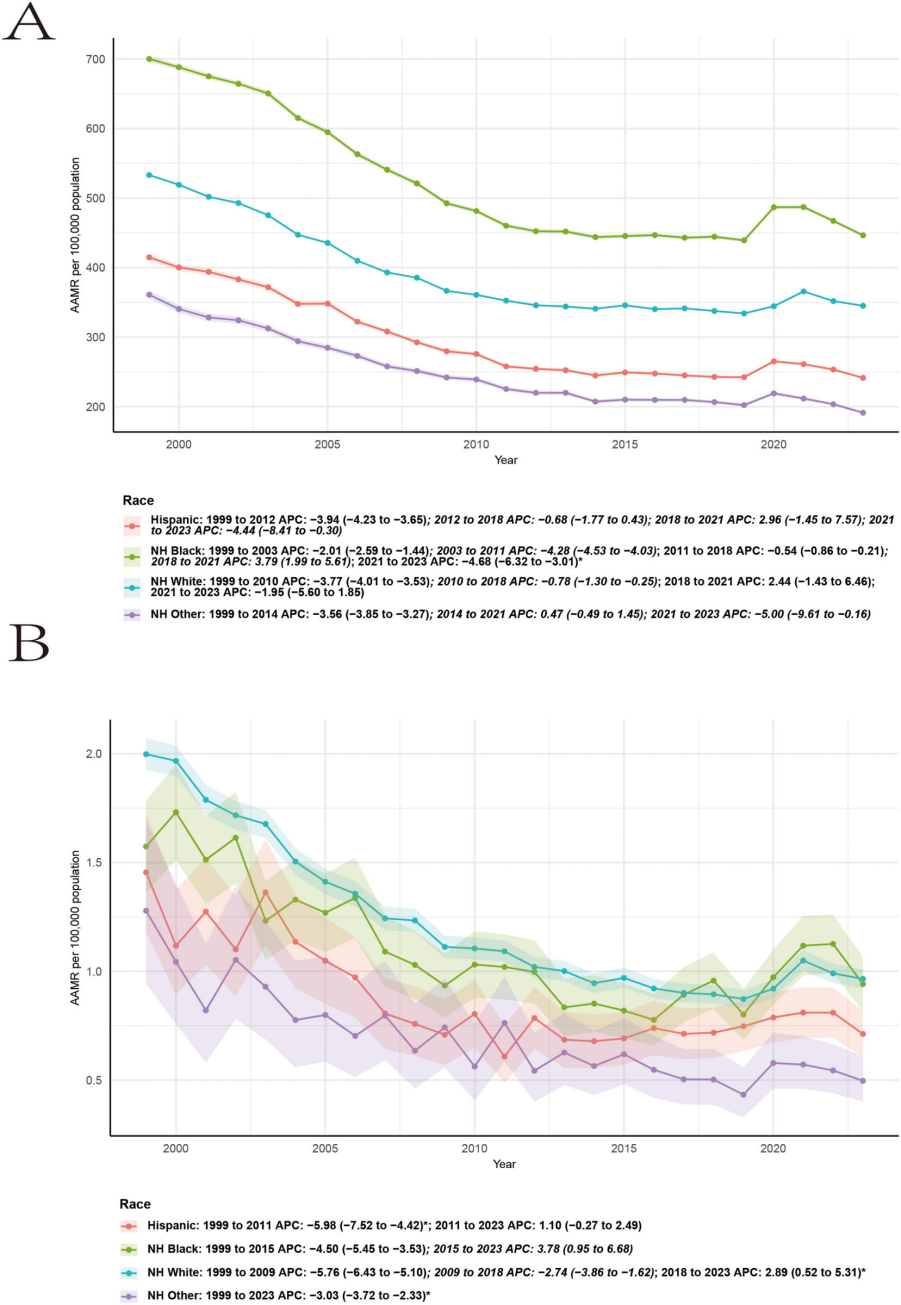
The age-adjusted mortality rate per 10,000 people for CVD **(A)** and RA-related CVD **(B)**, stratified by race, in the United States from 1999 to 2023.

Racial and ethnic stratification revealed marked disparities in RA-related cardiovascular mortality (Figure 5; Tables 1, 2 and Supplementary Table S1). Non-Hispanic White individuals accounted for the largest number of deaths and demonstrated long-term declines in AAMRs, with evidence of slowing improvement in recent years. Non-Hispanic Black individuals consistently exhibited higher AAMRs compared with other racial and ethnic groups and showed less pronounced declines over time. Hispanic and other racial/ethnic populations had lower overall AAMRs, though temporal trends were more variable. Joinpoint regression analyses by race/ethnicity identified heterogeneous APCs across groups, indicating persistent disparities in both mortality levels and temporal trends.

## 4 Discussion

This study describes long-term temporal trends in RA-related cardiovascular disease (CVD) mortality among U.S. adults aged ≥25 years from 1999 to 2023 using the CDC WONDER database. Overall, RA-related CVD mortality declined substantially over the study period, with a negative average annual percent change (AAPC). However, joinpoint analyses identified a recent reversal characterized by increasing mortality after approximately 2017. This pattern suggests that earlier improvements in population-level mortality trends have plateaued or partially reversed in recent years, although the underlying drivers of this shift cannot be determined from mortality certificate data alone.

Previous epidemiological studies have consistently demonstrated excess cardiovascular mortality among individuals with rheumatoid arthritis compared with the general population, with reported standardized mortality ratios of approximately 2.5 (19). Prior work also documented a sustained decline in RA-related mortality through the mid-2010s, which aligns with the early downward trends observed in our analysis. Similarly, the Global Burden of Disease (GBD) 2021 study reported a 23.8% reduction in the global age-standardized mortality rate for RA between 1999 and 2021 (29). Extending these findings, our study demonstrates that this favorable long-term trend in the United States has not continued uniformly into more recent years and that mortality patterns vary substantially across demographic and geographic subgroups.

The observed increase in RA-related CVD mortality after 2017 should be interpreted cautiously. This temporal shift coincides with several broader contextual factors, including population aging, evolving death certificate coding practices, and the COVID-19 pandemic. Individuals with chronic inflammatory and immunocompromised conditions, such as RA, may have experienced disproportionate adverse effects during the pandemic due to disruptions in routine care, delayed cardiovascular evaluation, and increased susceptibility to severe infection. These factors may have contributed to recent mortality patterns, although causal inference is not possible within the scope of this study.

Pronounced demographic disparities were evident across sex, geography, and urban–rural residence. Although females accounted for a greater absolute number of RA-related CVD deaths, males consistently exhibited higher age-adjusted mortality rates (AAMRs), indicating a disproportionate cardiovascular mortality burden among men with RA. This finding is consistent with prior literature suggesting that male patients with RA experience greater cardiovascular comorbidity despite lower disease prevalence (30–32). Potential explanations include sex-specific differences in inflammatory burden, hormonal regulation, and baseline cardiovascular risk profiles, though these mechanisms warrant further investigation (33, 34).

Geographic heterogeneity was also observed. While all U.S. Census regions experienced substantial declines in RA-related CVD mortality during the early study period, the Southern region demonstrated a notable increase beginning in the mid-2010s, contributing to the recent national-level reversal. These regional patterns may reflect variation in socioeconomic conditions, cardiovascular risk factor prevalence, healthcare access, and availability of specialized rheumatologic and cardiovascular services. In addition, non-metropolitan areas exhibited higher AAMRs than metropolitan areas throughout much of the study period. This disparity may be influenced by structural barriers to care, including limited healthcare infrastructure and reduced access to specialty services, rather than differences in disease biology alone (35, 36).

Substantial racial and ethnic disparities were also observed. Non-Hispanic White individuals accounted for the largest number of deaths, likely reflecting population size and RA prevalence. Among non-Hispanic Black individuals, mortality declined through approximately 2015 before increasing in subsequent years, a pattern that may be influenced by persistent structural inequities, socioeconomic disadvantage, and barriers to accessing timely rheumatologic and cardiovascular care (37, 38). Hispanic populations exhibited a more complex trajectory, characterized by periods of decline and increase. This pattern may partially reflect changes associated with acculturation, evolving cardiovascular risk profiles, and heterogeneous access to healthcare services, often described within the context of the “Hispanic paradox” (18, 39).

Overall, this study provides a descriptive overview of long-term trends and disparities in RA-related CVD mortality in the United States. The findings identify population subgroups with disproportionately high mortality and underscore the need for further research to clarify the biological, social, and healthcare-related mechanisms underlying these disparities, rather than implying direct deficiencies in clinical management or treatment effectiveness.

### 4.1 Limitation

Several limitations should be considered when interpreting these findings. First, this analysis relied on death certificate data from the CDC WONDER database, and misclassification or underreporting of rheumatoid arthritis and cardiovascular disease is possible. Second, the database lacks detailed information on disease severity, laboratory markers, treatment regimens, and socioeconomic variables, precluding adjustment for important confounders. Third, because this study is descriptive in nature, causal relationships between RA and cardiovascular mortality cannot be inferred. Finally, urban–rural trend analyses were limited to 2020 due to incomplete data availability for subsequent years.

## 5 Conclusion

Between 1999 and 2023, RA-related cardiovascular disease (CVD) mortality in the United States demonstrated an overall declining trend, followed by a recent period of stabilization and partial reversal. Pronounced disparities were observed across demographic and geographic subgroups, including differences by race/ethnicity, sex, urban–rural status, and region. These findings provide a descriptive overview of long-term patterns in RA-related CVD mortality and identify population subgroups with disproportionately high mortality. The observed trends likely reflect the combined influence of multiple factors, including population structure, healthcare access, and broader public health conditions. Further research is warranted to clarify the biological, social, and healthcare-related mechanisms underlying these disparities and recent trend changes, rather than implying direct deficiencies in clinical management.

## Data Availability

The original contributions presented in the study are included in the article/Supplementary Material, further inquiries can be directed to the corresponding author.

## References

[1] Helmick CG Felson DT Lawrence RC Gabriel S Hirsch R Kwoh CK. Estimates of the prevalence of arthritis and other rheumatic conditions in the United States. Part I. Arthritis Rheum. (2008) 58(1):15–25. 10.1002/art.23177 18163481

[2] Roth GA Johnson C Abajobir A Abd-Allah F Abera SF Abyu G. Global, regional, and national burden of cardiovascular diseases for 10 causes, 1990 to 2015. J Am Coll Cardiol. (2017) 70(1):1–25. 10.1016/j.jacc.2017.04.052 28527533 PMC5491406

[3] Almutairi K Nossent J Preen D Keen H Inderjeeth C. The global prevalence of rheumatoid arthritis: a meta-analysis based on a systematic review. Rheumatol Int. (2021) 41(5):863–77. 10.1007/s00296-020-04731-0 33175207

[4] Peterson E Gallagher MK Wilbur J. Rheumatoid arthritis: diagnosis and management for the family physician. Am Fam Physician. (2024) 110(5):515–26. 39556634

[5] Hitchon CA ONeil L Peschken CA Robinson DB Fowler-Woods A El-Gabalawy HS. Disparities in rheumatoid arthritis outcomes for north American indigenous populations. Int J Circumpolar Health. (2023) 82(1):2166447. 10.1080/22423982.2023.2166447 36642913 PMC9848324

[6] McDougall C Hurd K Barnabe C. Systematic review of rheumatic disease epidemiology in the indigenous populations of Canada, the United States, Australia, and New Zealand. Semin Arthritis Rheum. (2017). Philadelphia: W.B. Saunders Co-Elsevier Inc. 10.1016/j.semarthrit.2016.10.010 27914688

[7] Crowson CS Liao KP. Davis JM 3rd Solomon DH Matteson EL Knutson KL. Rheumatoid arthritis and cardiovascular disease. Am Heart J. (2013) 166(4):622–628.e1. 10.1016/j.ahj.2013.07.010 24093840 PMC3890244

[8] Dadoun S Zeboulon-Ktorza N Combescure C Elhai M Rozenberg S Gossec L. Mortality in rheumatoid arthritis over the last fifty years: systematic review and meta-analysis. Joint Bone Spine. (2013) 80(1):29–33. 10.1016/j.jbspin.2012.02.005 22459416

[9] Smolen JS Aletaha D McInnes IB. Rheumatoid arthritis. Lancet. (2016) 388(10055):2023–38. 10.1016/S0140-6736(16)30173-8 27156434

[10] Kadier K Dilixiati D Zhang X Li H Kuang L Huang J. Rheumatoid arthritis increases the risk of heart failure: results from the cross-sectional study in the US population and Mendelian randomization analysis in the European population. Front Immunol. (2024) 15:1377432. 10.3389/fimmu.2024.1377432 38863716 PMC11165030

[11] Semb AG Ikdahl E Wibetoe G Crowson C Rollefstad S. Atherosclerotic cardiovascular disease prevention in rheumatoid arthritis. Nat Rev Rheumatol. (2020) 16(7):361–79. 10.1038/s41584-020-0428-y 32494054

[12] Kalogeropoulos A Georgiopoulou V Psaty BM Rodondi N Smith AL Harrison DG. Inflammatory markers and incident heart failure risk in older adults: the health ABC (health, aging, and body composition) Study. J Am Coll Cardiol. (2010) 55(19):2129–37. 10.1016/j.jacc.2009.12.045 20447537 PMC3267799

[13] Damen JA Hooft L Schuit E Debray TP Collins GS Tzoulaki I. Prediction models for cardiovascular disease risk in the general population: systematic review. Br Med J. (2016) 353:i2416. 10.1136/bmj.i2416 27184143 PMC4868251

[14] Symmons DP Gabriel SE. Epidemiology of CVD in rheumatic disease, with a focus on RA and SLE. Nat Rev Rheumatol. (2011) 7(7):399–408. 10.1038/nrrheum.2011.75 21629241

[15] Sun X Qian Y Cheng W Ye D Liu B Zhou D. Characterizing the polygenic overlap and shared loci between rheumatoid arthritis and cardiovascular diseases. BMC Med. (2024) 22(1):152. 10.1186/s12916-024-03376-1 38589871 PMC11003061

[16] Ogdie A Yu Y Haynes K Love TJ Maliha S Jiang Y. Risk of major cardiovascular events in patients with psoriatic arthritis, psoriasis and rheumatoid arthritis: a population-based cohort study. Ann Rheum Dis. (2015) 74(2):326–32. 10.1136/annrheumdis-2014-205675 25351522 PMC4341911

[17] Strait A Castillo F Choden S Li J Whitaker E Falasinnu T. Demographic characteristics of participants in rheumatoid arthritis randomized clinical trials: a systematic review. JAMA Netw Open. (2019) 2(11):e1914745. 10.1001/jamanetworkopen.2019.14745 31722023 PMC6902779

[18] Ciofoaia EI Pillarisetty A Constantinescu F. Health disparities in rheumatoid arthritis. Ther Adv Musculoskelet Dis. (2022) 14:1759720×221137127. 10.1177/1759720X221137127 PMC9677290 36419481

[19] Almutairi KB Inderjeeth CA Preen DB Keen HI Nossent JC. Mortality trends among patients with rheumatoid arthritis in western Australia. Rheumatol Ther. (2023) 10(4):1021–37. 10.1007/s40744-023-00562-0 37335433 PMC10326173

[20] Morales-Etchegaray I Garcia-Carrasco M Munguía-Realpozo P Mendoza-Pinto C Méndez-Martínez S Navarro-Milán O. Changing trends in rheumatoid arthritis mortality in Mexico, from 1998 to 2017. Rheumatol Int. (2021) 41(12):2225–31. 10.1007/s00296-021-05013-z 34609597

[21] Terry K Makhlouf M Altarabsheh SE Deo V Petermann-Rocha F Elgudin Y. Trends in cardiovascular disease mortality by county-level social vulnerability Index in the United States. J Am Heart Assoc. (2023) 12(20):e030290. 10.1161/JAHA.123.030290 37804196 PMC10757513

[22] Woodruff RC Tong X Khan SS Shah NS Jackson SL Loustalot F. Trends in cardiovascular disease mortality rates and excess deaths, 2010–2022. Am J Prev Med. (2024) 66(4):582–9. 10.1016/j.amepre.2023.11.009 37972797 PMC10957309

[23] Malik R Chauhan G Traylor M Sargurupremraj M Okada Y Mishra A. Multiancestry genome-wide association study of 520,000 subjects identifies 32 loci associated with stroke and stroke subtypes. Nat Genet. (2018) 50(4):524–37. 10.1038/s41588-018-0058-3 29531354 PMC5968830

[24] Vandenbroucke JP von Elm E Altman DG Gøtzsche PC Mulrow CD Pocock SJ. Strengthening the reporting of observational studies in epidemiology (STROBE): explanation and elaboration. PLoS Med. (2007) 4(10):e297. 10.1371/journal.pmed.0040297 17941715 PMC2020496

[25] Woods K Beidas M Murugan V Billion T Tauseef A Mirza M. Trends in influenza- and pneumonia-related mortality in lung cancer patients from 1999 to 2022: a retrospective CDC WONDER analysis. Respir Res. (2025) 26(1):267. 10.1186/s12931-025-03336-0 40890819 PMC12403505

[26] Wang Y Huang Y Antwi SO Taner CB Yang L. Racial disparities in liver disease mortality trends among black and white populations in the United States, 1999–2020: an analysis of CDC WONDER database. Am J Gastroenterol. (2024) 119(4):682–9. 10.14309/ajg.0000000000002561 37830524

[27] Siddiqi TJ Khan Minhas AM Greene SJ Van Spall HGC Khan SS Pandey A. Trends in heart failure-related mortality among older adults in the United States from 1999 to 2019. JACC Heart Fail. (2022) 10(11):851–9. 10.1016/j.jchf.2022.06.012 36328654

[28] Gao X Lv F He X Zhao Y Liu Y Zu J. Impact of the COVID-19 pandemic on liver disease-related mortality rates in the United States. J Hepatol. (2023) 78(1):16–27. 10.1016/j.jhep.2022.07.028 35988691 PMC9611810

[29] Black RJ Cross M Haile LM Culbreth GT Steinmetz JD Hagins H. Global, regional, and national burden of rheumatoid arthritis, 1990–2020, and projections to 2050: a systematic analysis of the global burden of disease study 2021. Lancet Rheumatol. (2023) 5(10):e594–610. 10.1016/S2665-9913(23)00211-4 37795020 PMC10546867

[30] Maranini B Bortoluzzi A Silvagni E Govoni M. Focus on sex and gender: what we need to know in the management of rheumatoid arthritis. J Pers Med. (2022) 12(3):499. 10.3390/jpm12030499 35330498 PMC8948892

[31] Yu F Chen H Li Q Tao M Jin Z Geng L. Secular trend of mortality and incidence of rheumatoid arthritis in global,1990–2019: an age period cohort analysis and joinpoint analysis. BMC Pulm Med. (2023) 23(1):356. 10.1186/s12890-023-02594-2 37737172 PMC10515246

[32] van Vollenhoven RF. Sex differences in rheumatoid arthritis: more than meets the eye. BMC Med. (2009) 7:12. 10.1186/1741-7015-7-12 19331649 PMC2670321

[33] O'Brien SM Fitzgerald P Scully P Landers A Scott LV Dinan TG. Impact of gender and menstrual cycle phase on plasma cytokine concentrations. Neuroimmunomodulation. (2007) 14(2):84–90. 10.1159/000107423 17713355

[34] Cutolo M Sulli A Capellino S Villaggio B Montagna P Seriolo B. Sex hormones influence on the immune system: basic and clinical aspects in autoimmunity. Lupus. (2004) 13(9):635–8. 10.1191/0961203304lu1094oa 15485092

[35] Hoover K, Siemiatycki J, Jarret M, Abrahamowicz M, White W. Reanalysis of the Harvard Six Cities Study and the American Cancer Society Study of Particulate Air Pollution and Mortality: A Special Report of the Institute’s Particle Epidemiology Reanalysis Project. Cambridge MA: Health Effects Institute (2000).

[36] Abrams LR Myrskylä M Mehta NK. The growing rural-urban divide in US life expectancy: contribution of cardiovascular disease and other major causes of death. Int J Epidemiol. (2022) 50(6):1970–8. 10.1093/ije/dyab158 34999859 PMC8743112

[37] Kattamuri L Duggal S Aparece JP Sairam S. Cardiovascular risk factor and atherosclerosis in rheumatoid arthritis (RA). Curr Cardiol Rep. (2025) 27(1):31. 10.1007/s11886-025-02198-8 39831939

[38] Zhu C Shi T Jiang C Liu B Baldassarre LA Zarich S. Racial and ethnic disparities in all-cause and cardiovascular mortality among cancer patients in the U.S. JACC CardioOncol. (2023) 5(1):55–66. 10.1016/j.jaccao.2022.10.013 36875907 PMC9982284

[39] Liou KT Ashare R Worster B Jones KF Yeager KA Acevedo AM. SIO-ASCO guideline on integrative medicine for cancer pain management: implications for racial and ethnic pain disparities. JNCI Cancer Spectr. (2023) 7(4):pkad042. 10.1093/jncics/pkad042 37307074 PMC10336300

