# Correction: Trends in cardiovascular mortality related to rheumatoid arthritis among U.S. adults, 1999–2023

**DOI:** 10.3389/fcvm.2026.1865492

**Published:** 2026-06-09

**Authors:** Ye Jiang, Zhichao Meng, Huiqi Yuan, Guanghua Liang, Yongping Cao

**Affiliations:** 1 Department of Orthopedics, Peking University First Hospital, Beijing, China; 2 School of Public Health, Capital Medical University, Beijing, China

**Keywords:** cardiovascular disease, CDC WONDER, disease burden, epidemiological trends, rheumatoid arthritis

Correction on: Jiang Y, Meng Z, Yuan H, Liang G and Cao Y (2026) Trends in cardiovascular mortality related to rheumatoid arthritis among U.S. adults, 1999–2023. *Front. Cardiovasc. Med.* 13:1773739. doi: 10.3389/fcvm.2026.1773739

In the original article, the caption for Figure 3 was incorrectly published as:

“The age-adjusted mortality rate per 10,000 people due to CVD (A) and RA-related CVD (B) in the United States, stratified by race, from 1999 to 2023.”

The corrected caption for Figure 3 is:

“The age-adjusted mortality rate per 10,000 people for CVD (A) and RA-related CVD (B), stratified by gender, in the United States from 1999 to 2023”

In the original article, the caption for Figure 4 was incorrectly published as:

“The age-adjusted mortality rate of CVD (A) and RA-related CVD (B) per 10,000 people by gender in the United States from 1999 to 2023.”

The corrected caption of Figure 4 is:

“Deaths related to CVD (A) and RA-related CVD (B) among U.S. adults, stratified by urbanization, from 1999 to 2020.”

In the original article, the caption for Figure 5 was incorrectly published as:

“Caption Deaths related to CVD (A) and RA-related CVD (B) among adult Americans, grouped by urbanization, from 1999 to 2020. ”

The corrected caption of Figure 5 is:

“The age-adjusted mortality rate per 10,000 people for CVD (A) and RA-related CVD (B), stratified by race, in the United States from 1999 to 2023.”

The original version of this article has been updated.

