# Supplementary material for: Trends in cardiovascular mortality related to rheumatoid arthritis among U.S. adults, 1999–2023
Source: Front Cardiovasc Med. 2026 Mar 23;13:1773739. doi: 10.3389/fcvm.2026.1773739 (PMC13050709; doi:10.3389/fcvm.2026.1773739)
Supplement: Supplementary file 1 [file Table1.docx]

| Year | Female | | Male | | Northeast | | Midwest | | South | | West | | Hispanic | | NH Black | | NH White | | NH Other | |
| --- | --- | --- | --- | --- | --- | --- | --- | --- | --- | --- | --- | --- | --- | --- | --- | --- | --- | --- | --- | --- |
|  | Deaths | AAMR | Deaths | AAMR | Deaths | AAMR | Deaths | AAMR | Deaths | AAMR | Deaths | AAMR | Deaths | AAMR | Deaths | AAMR | Deaths | AAMR | Deaths | AAMR |
| 1999 | 2539 | 2.328（2.237，2.419） | 861 | 1.236（1.152，1.320） | 586 | 1.552（1.426，1.678） | 939 | 2.210（2.069，2.352） | 1,089 | 1.750（1.646，1.855） | 786 | 2.263（2.104，2.422） | 118 | 1.455（1.182，1.727） | 219 | 1.574（1.364，1.784） | 3,004 | 1.998（1.926，2.069） | 52 | 1.278（0.946，1.690） |
| 2000 | 2486 | 2.258（2.169，2.348） | 888 | 1.259（1.174，1.344） | 619 | 1.637（1.508，1.767） | 931 | 2.164（2.025，2.304） | 1,059 | 1.684（1.583，1.786） | 765 | 2.157（2.004，2.311） | 93 | 1.117（0.895，1.378） | 247 | 1.731（1.512，1.950） | 2,980 | 1.967（1.896，2.038） | 47 | 1.043（0.758，1.401） |
| 2001 | 2343 | 2.096（2.011，2.182） | 809 | 1.135（1.055，1.215） | 542 | 1.402（1.284，1.521） | 880 | 2.035（1.901，2.170） | 1,007 | 1.584（1.486，1.682） | 723 | 2.023（1.875，2.171） | 113 | 1.274（1.030，1.518） | 223 | 1.513（1.311，1.714） | 2,768 | 1.788（1.721，1.855） | 40 | 0.820（0.581，1.126） |
| 2002 | 2288 | 2.029（1.945，2.112） | 788 | 1.089（1.011，1.166） | 538 | 1.373（1.257，1.489） | 839 | 1.934（1.803，2.065） | 988 | 1.520（1.425，1.615） | 711 | 1.913（1.772，2.054） | 99 | 1.100（0.888，1.348） | 234 | 1.614（1.404，1.823） | 2,680 | 1.717（1.652，1.782） | 54 | 1.053（0.781，1.388） |
| 2003 | 2220 | 1.959（1.876，2.041） | 783 | 1.062（0.986，1.138） | 514 | 1.287（1.175，1.399） | 816 | 1.846（1.719，1.973） | 1,002 | 1.540（1.444，1.636） | 671 | 1.776（1.641，1.911） | 128 | 1.363（1.120，1.605） | 183 | 1.232（1.051，1.412） | 2,632 | 1.677（1.613，1.741） | 54 | 0.929（0.687，1.228） |
| 2004 | 2098 | 1.802（1.724，1.880） | 687 | 0.902（0.833，0.970） | 459 | 1.136（1.031，1.241） | 765 | 1.697（1.576，1.817） | 886 | 1.320（1.233，1.408） | 675 | 1.755（1.622，1.887） | 119 | 1.136（0.923，1.349） | 204 | 1.329（1.144，1.514） | 2,416 | 1.504（1.444，1.565） | 44 | 0.776（0.559，1.049） |
| 2005 | 2007 | 1.732（1.655，1.809） | 671 | 0.858（0.792，0.924） | 482 | 1.185（1.078，1.291） | 734 | 1.616（1.499，1.733） | 839 | 1.223（1.140，1.307） | 623 | 1.576（1.451，1.701） | 117 | 1.048（0.851，1.245） | 198 | 1.269（1.089，1.449） | 2,305 | 1.412（1.354，1.469） | 50 | 0.800（0.585，1.066） |
| 2006 | 1905 | 1.607（1.534，1.680） | 678 | 0.844（0.780，0.909） | 402 | 1.001（0.903，1.100） | 712 | 1.540（1.427，1.654） | 838 | 1.181（1.101，1.262） | 631 | 1.544（1.423，1.665） | 114 | 0.972（0.788，1.157） | 211 | 1.337（1.153，1.521） | 2,204 | 1.357（1.300，1.414） | 49 | 0.703（0.513，0.941） |
| 2007 | 1781 | 1.463（1.394，1.532） | 621 | 0.771（0.710，0.833） | 414 | 1.000（0.903，1.097） | 675 | 1.447（1.338，1.557） | 782 | 1.110（1.032，1.188） | 531 | 1.275（1.166，1.384） | 100 | 0.806（0.644，0.969） | 180 | 1.091（0.927，1.254） | 2,068 | 1.244（1.190，1.297） | 53 | 0.797（0.593，1.048） |
| 2008 | 1819 | 1.499（1.429，1.568） | 576 | 0.683（0.626，0.740） | 394 | 0.946（0.852，1.040） | 679 | 1.439（1.330，1.547） | 762 | 1.061（0.985，1.138） | 560 | 1.309（1.200，1.418） | 99 | 0.758（0.611，0.930） | 176 | 1.030（0.874，1.185） | 2,067 | 1.234（1.180，1.287） | 48 | 0.635（0.463，0.850） |
| 2009 | 1636 | 1.301（1.237，1.365） | 571 | 0.681（0.625，0.738） | 368 | 0.885（0.793，0.976） | 603 | 1.247（1.146，1.347） | 689 | 0.920（0.851，0.990） | 547 | 1.225（1.122，1.329） | 95 | 0.709（0.570，0.871） | 165 | 0.934（0.788，1.081） | 1,888 | 1.112（1.062，1.163） | 58 | 0.743（0.558，0.969） |
| 2010 | 1667 | 1.304（1.241，1.368） | 586 | 0.678（0.622，0.734） | 356 | 0.832（0.745，0.919） | 576 | 1.187（1.089，1.285） | 749 | 0.982（0.911，1.053） | 572 | 1.274（1.168，1.379） | 110 | 0.804（0.649，0.959） | 183 | 1.031（0.878，1.184） | 1,910 | 1.105（1.055，1.155） | 47 | 0.562（0.407，0.758） |
| 2011 | 1665 | 1.280（1.217，1.342） | 610 | 0.664（0.610，0.717） | 377 | 0.844（0.757，0.931） | 598 | 1.204（1.106，1.302） | 716 | 0.904（0.837，0.971） | 584 | 1.256（1.153，1.359） | 91 | 0.608（0.487，0.750） | 189 | 1.020（0.871，1.170） | 1,928 | 1.092（1.043，1.141） | 66 | 0.763（0.586，0.976） |
| 2012 | 1620 | 1.207（1.147，1.267） | 617 | 0.668（0.615，0.722） | 339 | 0.755（0.673，0.837） | 595 | 1.169（1.073，1.264） | 721 | 0.883（0.818，0.948） | 582 | 1.195（1.096，1.293） | 126 | 0.785（0.644，0.926） | 193 | 0.997（0.852，1.143） | 1,865 | 1.020（0.973，1.067） | 52 | 0.543（0.402，0.718） |
| 2013 | 1570 | 1.160（1.101，1.218） | 639 | 0.668（0.616，0.721） | 356 | 0.779（0.696，0.861） | 557 | 1.087（0.995，1.178） | 717 | 0.869（0.805，0.934） | 579 | 1.166（1.069，1.262） | 113 | 0.686（0.556，0.815） | 164 | 0.834（0.704，0.965） | 1,868 | 1.001（0.955，1.047） | 62 | 0.627（0.477，0.809） |
| 2014 | 1561 | 1.126（1.070，1.183） | 564 | 0.578（0.530，0.627） | 346 | 0.755（0.673，0.837） | 569 | 1.089（0.998，1.179） | 635 | 0.738（0.680，0.796） | 575 | 1.129（1.034，1.224） | 128 | 0.679（0.556，0.801） | 175 | 0.852（0.722，0.982） | 1,758 | 0.945（0.901，0.990） | 63 | 0.565（0.432，0.725） |
| 2015 | 1614 | 1.138（1.081，1.195） | 589 | 0.565（0.518，0.612） | 319 | 0.685（0.608，0.761） | 569 | 1.067（0.978，1.156） | 711 | 0.804（0.745，0.864） | 604 | 1.172（1.077，1.267） | 132 | 0.692（0.570，0.813） | 175 | 0.819（0.695，0.944） | 1,819 | 0.970（0.924，1.015） | 70 | 0.619（0.481，0.785） |
| 2016 | 1610 | 1.107（1.052，1.162） | 554 | 0.529（0.484，0.573） | 335 | 0.714（0.635，0.792） | 537 | 0.994（0.909，1.079） | 730 | 0.792（0.734，0.850） | 562 | 1.033（0.946，1.119） | 145 | 0.739（0.615，0.862） | 178 | 0.777（0.658，0.895） | 1,773 | 0.921（0.878，0.965） | 63 | 0.547（0.419，0.703） |
| 2017 | 1628 | 1.089（1.035，1.142） | 600 | 0.568（0.521，0.614） | 334 | 0.682（0.607，0.756） | 554 | 1.006（0.921，1.091） | 757 | 0.800（0.742，0.857） | 583 | 1.029（0.944，1.113） | 153 | 0.713（0.596，0.829） | 205 | 0.893（0.766，1.019） | 1,798 | 0.901（0.859，0.943） | 68 | 0.504（0.389，0.642） |
| 2018 | 1629 | 1.079（1.026，1.132） | 620 | 0.562（0.517，0.607） | 346 | 0.694（0.620，0.768） | 568 | 1.005（0.922，1.089） | 783 | 0.821（0.763，0.879） | 552 | 0.960（0.879，1.041） | 159 | 0.718（0.604，0.833） | 226 | 0.957（0.829，1.086） | 1,795 | 0.894（0.853，0.936） | 64 | 0.502（0.386，0.643） |
| 2019 | 1592 | 1.031（0.980，1.083） | 639 | 0.565（0.521，0.610） | 343 | 0.674（0.601，0.748） | 558 | 0.971（0.889，1.053） | 733 | 0.744（0.689，0.799） | 597 | 1.014（0.931，1.097） | 173 | 0.747（0.633，0.861） | 194 | 0.802（0.685，0.918） | 1,797 | 0.873（0.832，0.913） | 64 | 0.433（0.330，0.557） |
| 2020 | 1774 | 1.119（1.066，1.171） | 656 | 0.574（0.529，0.618） | 372 | 0.727（0.652，0.802） | 617 | 1.045（0.962，1.129） | 811 | 0.795（0.739，0.850） | 630 | 1.035（0.953，1.117） | 194 | 0.788（0.673，0.902） | 243 | 0.973（0.847，1.099） | 1,906 | 0.920（0.878，0.962） | 85 | 0.579（0.461，0.717） |
| 2021 | 1,848 | 1.257（1.198，1.316） | 737 | 0.650（0.602，0.698） | 409 | 0.827（0.746，0.909） | 618 | 1.130（1.039，1.220） | 940 | 0.978（0.914，1.042） | 618 | 1.053（0.968，1.137） | 197 | 0.811（0.694，0.927） | 274 | 1.118（0.980，1.256） | 2,013 | 1.049（1.003，1.096） | 96 | 0.571（0.461，0.701） |
| 2022 | 1,928 | 1.212（1.157，1.266） | 712 | 0.610（0.564，0.656） | 412 | 0.799（0.721，0.877） | 607 | 1.039（0.955，1.122） | 969 | 0.924（0.865，0.983） | 652 | 1.071（0.988，1.154） | 203 | 0.810（0.696，0.924） | 285 | 1.126（0.992，1.260） | 2,048 | 0.991（0.947，1.034） | 99 | 0.544（0.441，0.665） |
| 2023 | 1,835 | 1.141（1.089，1.194） | 687 | 0.566（0.522，0.610） | 365 | 0.705（0.632，0.778） | 595 | 1.010（0.928，1.092） | 909 | 0.866（0.808，0.923） | 653 | 1.029（0.949，1.109） | 193 | 0.712（0.607，0.818） | 236 | 0.942（0.818，1.065） | 1,993 | 0.965（0.922，1.008） | 96 | 0.497（0.401，0.609） |
|  | 46,663 |  | 16743 |  | 10327 |  | 16691 |  | 20,822 |  | 15566 |  | 3312 |  | 5160 |  | 53,283 |  | 1544 |  |
| Year | Metropolitan | | Nonmetropolitan | |  |  |  |  |  |  |  |  |  |  |  |  |  |  |  |  |
|  | Deaths | AAMR | Deaths | AAMR |  |  |  |  |  |  |  |  |  |  |  |  |  |  |  |  |
| 1,999 | 2,633 | 1.839（1.769，1.910） | 767 | 2.257（2.097，2.416） |  |  |  |  |  |  |  |  |  |  |  |  |  |  |  |  |
| 2,000 | 2,590 | 1.771（1.702，1.839） | 784 | 2.298（2.137，2.459） |  |  |  |  |  |  |  |  |  |  |  |  |  |  |  |  |
| 2,001 | 2,409 | 1.628（1.562，1.693） | 743 | 2.159（2.003，2.314） |  |  |  |  |  |  |  |  |  |  |  |  |  |  |  |  |
| 2,002 | 2,336 | 1.566（1.502，1.630） | 740 | 2.130（1.976，2.284） |  |  |  |  |  |  |  |  |  |  |  |  |  |  |  |  |
| 2,003 | 2,341 | 1.520（1.458，1.582） | 662 | 1.901（1.756，2.046） |  |  |  |  |  |  |  |  |  |  |  |  |  |  |  |  |
| 2,004 | 2,176 | 1.399（1.340，1.458） | 609 | 1.711（1.575，1.848） |  |  |  |  |  |  |  |  |  |  |  |  |  |  |  |  |
| 2,005 | 2,029 | 1.274（1.218，1.329） | 649 | 1.807（1.668，1.947） |  |  |  |  |  |  |  |  |  |  |  |  |  |  |  |  |
| 2,006 | 1,999 | 1.233（1.179，1.287） | 584 | 1.600（1.470，1.731） |  |  |  |  |  |  |  |  |  |  |  |  |  |  |  |  |
| 2,007 | 1,825 | 1.092（1.041，1.142） | 577 | 1.587（1.457，1.717） |  |  |  |  |  |  |  |  |  |  |  |  |  |  |  |  |
| 2,008 | 1,789 | 1.058（1.009，1.108） | 606 | 1.646（1.513，1.779） |  |  |  |  |  |  |  |  |  |  |  |  |  |  |  |  |
| 2,009 | 1,712 | 1.009（0.961，1.058） | 495 | 1.307（1.191，1.423） |  |  |  |  |  |  |  |  |  |  |  |  |  |  |  |  |
| 2,010 | 1,730 | 0.988（0.941，1.035） | 523 | 1.403（1.280，1.525） |  |  |  |  |  |  |  |  |  |  |  |  |  |  |  |  |
| 2,011 | 1,807 | 1.000（0.954，1.047） | 468 | 1.214（1.104，1.325） |  |  |  |  |  |  |  |  |  |  |  |  |  |  |  |  |
| 2,012 | 1,749 | 0.955（0.910，1.000） | 488 | 1.248（1.137，1.359） |  |  |  |  |  |  |  |  |  |  |  |  |  |  |  |  |
| 2,013 | 1,720 | 0.901（0.858，0.945） | 489 | 1.222（1.113，1.331） |  |  |  |  |  |  |  |  |  |  |  |  |  |  |  |  |
| 2,014 | 1,661 | 0.847（0.806，0.889） | 464 | 1.143（1.039，1.248） |  |  |  |  |  |  |  |  |  |  |  |  |  |  |  |  |
| 2,015 | 1,711 | 0.855（0.814，0.896） | 492 | 1.203（1.094，1.312） |  |  |  |  |  |  |  |  |  |  |  |  |  |  |  |  |
| 2,016 | 1,721 | 0.830（0.790，0.870） | 443 | 1.056（0.957，1.155） |  |  |  |  |  |  |  |  |  |  |  |  |  |  |  |  |
| 2,017 | 1,747 | 0.822（0.783，0.861） | 481 | 1.124（1.023，1.226） |  |  |  |  |  |  |  |  |  |  |  |  |  |  |  |  |
| 2,018 | 1,821 | 0.846（0.807，0.886） | 428 | 0.990（0.895，1.085） |  |  |  |  |  |  |  |  |  |  |  |  |  |  |  |  |
| 2,019 | 1,755 | 0.791（0.754，0.829） | 476 | 1.082（0.983，1.181） |  |  |  |  |  |  |  |  |  |  |  |  |  |  |  |  |
| 2,020 | 1,934 | 0.858（0.819，0.896） | 496 | 1.110（1.010，1.209） |  |  |  |  |  |  |  |  |  |  |  |  |  |  |  |  |
|  | 43,195 |  | 12464 |  |  |  |  |  |  |  |  |  |  |  |  |  |  |  |  |  |

Supplementary table 1. The number of deaths and AAMR related to rheumatoid arthritis in different groups.
